# High ratio of pCXCR4/CXCR4 tumor infiltrating immune cells in primary high grade ovarian cancer is indicative for response to chemotherapy

**DOI:** 10.1186/s12885-022-09374-x

**Published:** 2022-04-09

**Authors:** Fabio Walther, Jana Ladina Berther, Alexandros Lalos, Michaela Ramser, Simone Eichelberger, Robert Mechera, Savas Soysal, Simone Muenst, Alberto Posabella, Uwe Güth, Sylvia Stadlmann, Luigi Terracciano, Raoul A. Droeser, Jasmin Zeindler, Gad Singer

**Affiliations:** 1 University Center for Gastrointestinal and Liver Diseases (Clarunis), University of Basel, Spitalstrasse 21, 4031 Basel, Switzerland; 2 Institute of Pathology, University Hospital Basel, Schönbeinstrasse 40, 4031 Basel, Switzerland; 3 Brustzentrum Zürich, Seefeldstrasse 214, 8008 Zürich, Switzerland; 4 Department of Gynecology and Obstetrics, University Hospital Basel, Spitalstrasse 21, 4031 Basel, Switzerland; 5 Institute of Pathology, Kantonsspital Baden AG, Im Ergel 1, 5404 Baden, Switzerland

**Keywords:** Ovarian cancer, CXCR4, PCXCR4, Tumor expression, Tumor infiltrating immune cells, Prognostic significance, Chemokines, Chemosensitivity

## Abstract

**Background:**

Ovarian cancer (OC) is the fifth most common malignant female cancer with a high mortality, mainly because of aggressive high-grade serous carcinomas (HGSOC), but also due to absence of specific early symptoms and effective detection strategies. The CXCL12-CXCR4 axis is considered to have a prognostic impact and to serve as potential therapeutic target. Therefore we investigated the role of pCXCR4 and CXCR4 expression of the tumor cells and of tumor infiltrating immune cells (TIC) in high-grade serous OC and their association with the recurrence-free (RFS) and overall survival (OS).

**Methods:**

A tissue microarray of 47 primary high grade ovarian serous carcinomas and their recurrences was stained with primary antibodies directed against CXCR4 and pCXCR4. Beside the evaluation of the absolute tumor as well as TIC expression in primary and recurrent cancer biopsies the corresponding ratios for pCXCR4 and CXCR4 were generated and analyzed. The clinical endpoints were response to chemotherapy, OS as well as RFS.

**Results:**

Patients with a high pCXCR4/CXCR4 TIC ratio in primary cancer biopsies showed a significant longer RFS during the first two years (*p* = 0.025). However, this effect was lost in the long-term analysis including a follow-up period of 5 years (*p* = 0.128). Interestingly, the Multivariate Cox regression analysis showed that a high pCXCR4/CXCR4 TIC ratio in primary cancer independently predicts longer RFS (HR 0.33; 95CI 0.13 - 0.81; *p* = 0.015). Furthermore a high dichotomized distribution of CXCR4 positive tumor expression in recurrent cancer biopsies showed a significantly longer 6-month RFS rate (*p* = 0.018) in comparison to patients with low CXCR4 positive tumor expression. However, this effect was not independent of known risk factors in a Multivariate Cox regression (HR 0.57; 95CI 0.24 - 1.33; *p* = 0.193).

**Conclusions:**

To the best of our knowledge we show for the first time that a high pCXCR4/CXCR4 TIC ratio in primary HGSOC biopsies is indicative for better RFS and response to chemotherapy.

**Highlights:**

• We observed a significant association between high pCXCR4/CXCR4 TIC ratio and better RFS in primary cancer biopsies, especially during the early postoperative follow-up and independent of known risk factors for recurrence.

• High CXCR4 tumor expression in recurrent HGSOC biopsies might be indicative for sensitivity to chemotherapy. We found evidence that at the beginning of the disease (early follow-up) the role of the immune response seems to be the most crucial factor for progression. On the other hand in recurrent/progressive disease the biology of the tumor itself becomes more important for prognosis.

• We explored for the first time the predictive and prognostic role of pCXCR4/CXCR4 TIC ratio in high-grade serous ovarian cancer.

## Introduction

Ovarian cancer (OC) is the fifth most common malignant cancer among women worldwide and accounts for 5% of all cancer-related deaths [1]. While ovarian cancer mortality has decreased by greater than 30% since the mid-1970s, still less than one-half of women survive beyond five years after diagnosis, mainly because of the predominance of the aggressive tumor biology of high-grade serous ovarian carcinoma (HGSOC) and the absence of specific early symptoms and effective early detection strategies [2]. Several studies have investigated the diagnostic, prognostic and therapeutic information of different biomarkers to improve early HGSOC detection and HGSOC treatment [3–6]. In a recent study we could show that high density of CD66b in primary high-grade ovarian cancer independently predicts response to chemotherapy [7].

HGSOC is a histopathological diagnosis, based on the evaluation of tissue following surgical removal. The diagnosis may also be based on tissue or fluid obtained via image-guided biopsy, paracentesis or thoracentesis, but less frequently. For most women with OC, the management approach used is surgical staging and cytoreduction followed by adjuvant chemotherapy [8]. Staging is performed surgically after histopathological confirmation of the disease, and also the operative macroscopic findings prior to the debulking determine the stage. This can be modified by histopathological as well as clinical or radiological evaluation [9]. Most women require adjuvant treatment, specifically those with stage IC or II disease; but patients with stage III or IV disease are also candidates for primary surgical cytoreduction followed by chemotherapy. Patients with clear cell histology or HGSOC are depending on the staging also candidates for adjuvant/additive chemotherapy [8].

CXCR4 is a 7-transmembrane G-protein coupled receptor [10] and highly expressed in a variety of cell types, including lymphocytes, endothelial, epithelial and hematopoietic stem cells, stromal fibroblasts and cancer cells [11] and plays a major role in embryogenesis, homeostasis and inflammation [12]. The receptor CXCR4 together with one of its chemokines, CXCL12 (also known as SDF-1), have been shown to play a key role in the tumor-stromal communication affecting cancer growth, angiogenesis and metastasis formation [13]. We recently investigated the role of SDF-1 in colorectal cancer (CRC) and found that its prognostic role depends on the expression of other immunomarkers in the tumor microenvironment [14].

The existing data concerning the prognostic impact of CXCR4 expression are controversial. Several studies demonstrated that high CXCR4 expression has been linked to cancer progression and metastases in hematopoietic as well as in various non-hematopoietic malignancies [15–17]. Furthermore, CXCR4 expression has shown a significant prognostic impact in colon cancer, primary cutaneous melanoma, B-ALL and in patients with breast cancer [18–21]. CXCR4 is expressed in various different tumor types and has been considered the most widely expressed chemokine receptor in many cancers including ovarian tumors [22, 23]. Studies also showed the role of CXCR4 in crosstalk between tumor cells and their microenvironment [15]. There is also some evidence that the CXCL12-CXCR4 axis plays a role in ovarian cancer metastasis and that this axis is a key determinator of tumor initiation and intraperitoneal metastasis in ovarian cancer [13, 23, 24].

However, the evidence regarding the prognostic and predictive role of CXCR4 in HGSOC is vague. Some studies have shown that CXCL12 and CXCR4 were highly expressed in HGSOC [24]. For instance, Kajiyama et al. suggested that there may be a link between the SDF-1/CXCR4 axis and enhanced intraperitoneal dissemination of epithelial ovarian cancer (EOC) and that CXCR4 may be a novel target for the treatment of EOC [25]. While, Jiang et al. provided the first evidence that CXCR4 expression could be an independent prognostic factor for epithelial ovarian cancer patients [26]. Moreover, a higher positive rate of CXCL12 and CXCR4 was found in non-metastatic as well as in metastatic HGSOC tissue [24]. In addition, expression of CXCL12 and CXCR4 is increased in metastatic HGSOC tissue compared to non-metastatic HGSOC tissue [24]. In clear cell carcinoma of the ovary, a report indicates that high expression of CXCR4 is associated with shorter progression free survival [27], while in CRC it had no prognostic significance. Interestingly, expression of its phosphorylated, activated form (pCXCR4) is associated with a favorable clinical outcome and represents a favorable prognostic factor in CRC [28]. In breast cancer pCXCR4 seems to have a better prognostic value than CXCR4 [29], while Konoplev et al. have shown a prognostic impact only for pCXCR4 expression in adult patients with B-ALL [21].

To the best of our knowledge, the prognostic and predictive role of pCXCR4 alone and in relation to CXCR4 has not been explored in ovarian cancer, yet. Therefore, the aim of this study was to investigate the prognostic and predictive value of the expression of these two markers alone and in relation to each other regarding RFS, OS and response to chemotherapy, in primary and corresponding recurrent ovarian cancer biopsies in a cohort of patients with HGOSC.

## Materials and methods

Approval to this study was given by the local ethics committee EKNZ (Ethikkomission Nordwest- und Zentralschweiz).

### Patients and study design

Forty-seven unselected, non-consecutive, clinically annotated, primary high grade ovarian serous carcinomas and their recurrences were contained in our TMA platform. Pathologist confirmed the initial histopathological diagnosis in all cases. Recurrence was defined as confirmed tumor mass in radiological examination, biopsies or during secondary surgical procedures, some patients showed an elevation of CA-125 levels. In cases with tumor biopsies or surgical tumor removal, pathologists confirmed the histopathological diagnosis of recurrence. Time to recurrence of ≤ 6 months after completion of platinum-based chemotherapy was defined as chemoresistant and time to recurrence of > 6 months as chemosensitive [30]. OS, RFS and response to chemotherapy were the primary endpoints. The initially collected variables were mean age at diagnosis, tumor stage, chemotherapy cycles, tumor grade, histopathologic subtype, presence of residual disease and time to death. Manuscript and results is REMARK compliant as established in NCI-EORTC's "Reporting Recommendations for Tumor Marker Prognostic Studies" (J Clin Oncol 23:9067, 2005) and "Reporting Recommendations for Tumor Marker Prognostic Studies (REMARK): An Abridged Explanation and Elaboration" [31]. Time period was 1985-2003 [30]. Rational for the sample size is not applicable due to the retrospective character of this study. The assay method is described below.

### Tissue microarray construction

Tissue samples of HGSOC were available at the Pathology Biobank at the Pathology of Cantonal Hospitals of St. Gallen, Liestal and Baden and the University Hospital Basel. Formalin-fixed, paraffin-embedded (FFPE) tissue blocks were prepared according to standard procedures. Tissue cylinders with a diameter of 0.6 mm were punched from morphologically representative areas of each donor block and transferred to a recipient paraffin block (30 × 25mm), using a semi-automated tissue arrayer. Each punch was made from the center of the tumor to enable each TMA spot to include at least 50% tumor cells. Histopathological data were collected from the individual reports at pathology while survival and clinical data were collected from the database at the hospital. The study was performed according to the guidelines of the institutional review boards of the participating institutions in compliance with specific regard to ethical standards and patient confidentiality.

### Immunohistochemistry

**Fig. 1:**
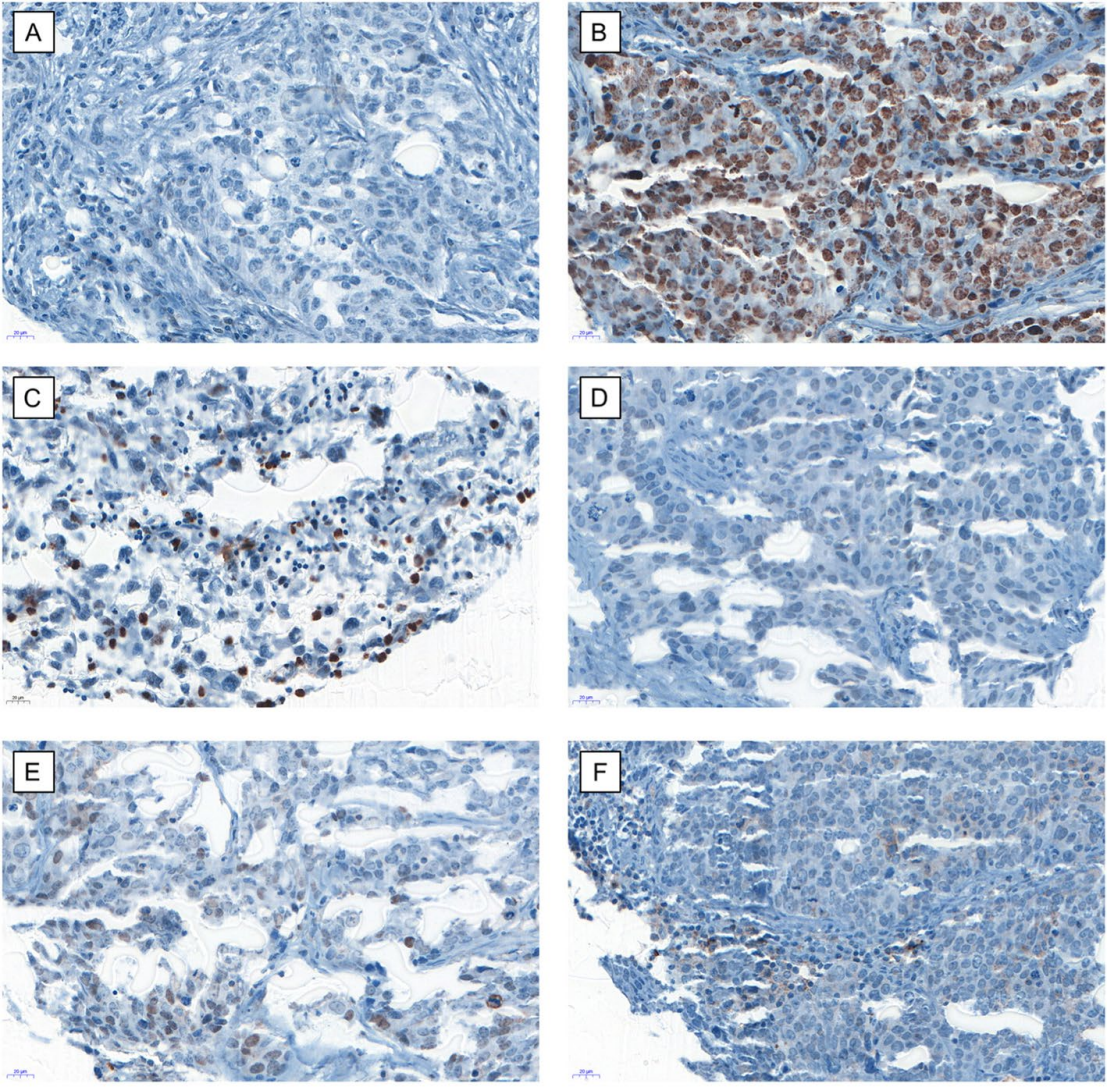
Example of staining in recurrent ovarian cancer biopsies. Example of low CXCR4 tumor expression (**A**), high CXCR4 tumor expression (**B**), high CXCR4 immune cell density (**C**), low pCXCR4 tumor expression (**D**), high pCXCR4 tumor expression (**E**) and high pCXCR4 immune cell density (**F**) in recurrent ovarian cancer biopsies, 40x

Primary antibodies directed against CXCR4 (Abcam, ab2074; 1:50) and pCXCR4 (Abcam, ab74012; 1:200) were used according to the staining protocols. We included positive control tissue samples. To perform immunohistochemistry, we used the automated staining system Benchmark XT (Roche/Ventana Medical Systems, Tuscon, AZ). A trained research fellow (J.L.B.) and a pathologist (G.S.) performed immunohistochemical evaluation blinded to clinical, survival and histopathological data. The immunohistochemical staining of CXCR4 and pS339-CXCR4 was established using paraffin-embedded Jurkat cell pellets known to express significant levels of CXCR4, which becomes phosphorylated on S339 upon phorbol-ester treatment [32]. Cells were scored per High Power Field (HPF). One TMA slide was used for each antibody. Antibodies were omitted for negative controls. There were single IHC stains. Statements were included in the methods section. Antibodies used for the different positive stainings are mentioned previously. The median was used to generate for cut-offs. Punches with no tissue and with division by zero in case of the ratio analysis were omitted and considered as random drop-outs. The stainings were performed as singles stainings. The cut-off value between high and low CXCR4 tumor expression in recurrent cancer biopsies was established by the median (84 cells/punch) which is the number of total cells in the tissue section. The cut-off value between high and low pCXCR4/CXCR4 TIC ratio in the overall cohort was set at 0.1875 using the median. Images of the evaluated punches can be seen in Fig. 1. Staining patterns for primary tumor tissue were similar to biopsies for recurrent cancer (data not shown).

### Statistical analysis

The Cox proportional Hazards regression model was used to explore associations with survival, while the used cut-off values for low or high pCXCR4/CXCR4 TIC ratio as well as CXCR4 tumor expression were obtained using median values, respectively. To determine the association of this ratio positivity with clinico-pathological features, Chi-square, Fisher’s exact, and Kruskal-Wallis tests were used. Finally, spearman’s correlation analyses of CXCR4 TIC, CXCR4 tumor expression, pCXCR4 TIC, pCXCR4 tumor expression, MPO, IL-17, FOXP3, OX40 TIC, OX40 tumor expression and CD66b in primary as well as in recurrent ovarian carcinoma was performed. Data of the other immune markers were available from previous publications [4, 5, 7, 28]. Univariate survival analysis was performed by Kaplan-Meier and log rank test. We assumed any clinicopathological information to be missing at random. Finally, data from low or high pCXCR4/CXCR4 TIC ratio expression in primary cancer as well as low or high CXCR4 tumor expression in recurrent cancer were entered into a Multivariate Cox regression analysis and calculation for Hazard ratios (HR) were done with using 95% confidence intervals (CI) to determine prognostic effects on survival time. The following clinico-pathological features were used: Age, high vs low pCXCR4/CXCR4, TIC ratio, residual disese < 2cm, residual disease > 2cm, n of chemotherapy cycles, FIGO stage (IIIA-IV). We considered *p*-values < 0.05 as statistically significant. STATA software version 13 (StataCorp, College Station, TX, USA) was used to perform statistical analyses.

## Results

### Basic demographic characteristics

Tissues were collected and analyzed from a total of 47 patients. The median age of the 47 patients was 58 years (range 34 - 77). 97.9% were staged as FIGO IIIA or higher. The majority of the patients had a FIGO stage IIIC (68.2%; *n* = 32). 83% of the patients had 6 or more chemotherapy cycles. 63.9% of the patients had a residual disease (36.2% < 2cm and 27.7% > 2cm) while 34% showed no residual disease. The 6-month RFS was 53% (95% CI = 0.38 - 0.63) and the 3-year OS was 47% (95% CI = 0.29 - 0.63). All patients suffered from a recurrence and 30 patients died during the follow up period. All patients had a histopathological diagnosis of HGSOC. Patient characteristics are summarized in Table 1.

**Table 1:** Patient characteristics (*n *= 47)^a^

	***N***=47 (100%)
**Age (median, range)**	58 (34-77)
**FIGO stage**	
**II**	1 (2.1)
**IIIA**	1 (2.1)
**IIIB**	5 (10.6)
**IIIC**	32 (68.2)
**IV**	8 (17.0)
**Residual disease**	
**None**	16 (34.0)
**<2cm**	17 (36.2)
**>2cm**	13 (27.7)
**Numbers of chemotherapy cycles**	
**<6**	7 (14.9)
**6 or more**	39 (83.0)
**CS**^**b**^	33 (70.2)
**CR**^**b**^	14 (29.8)
**6-month recurrent free survival (95%CI)**^**c**^	0.53 (0.38-0.66)
**3-year overall survival (95%CI)**^**c**^	0.47 (0.29-0.63)

^a^missing clinicopathological information was assumed to be missing at random

^b^*CS* chemosensitive, *CR* chemoresistant,

^c^*RFS* recurrence-free survival, *OS* overall survival

### Patient characteristics according to pCXCR4/CXCR4 ratio of tumor infiltrating immune cells in primary cancer biopsies

Patient characteristics according to the dichotomized distribution of pCXCR4/CXC4 TIC ratio in primary cancer biopsies did not show any significant differences for FIGO stage, 6-month RFS rate and OS. The results according to the dichotomized distribution of pCXCR4/CXCR4 TIC ratio in primary cancer biopsies are summarized in Table 2.

**Table 2:** Patients' characteristics according to pCXCR4/CXCR4 TIC ratio in primary biopsies in the overall cohort^a^

	pCXCR4/CXCR4^**high**^	pCXCR4/CXCR4^**low**^	***p***-value
	***n***=18 (100%)	***n***=18 (100%)	
**Age (median, range)**	61.5 (47-73)	58 (34-69)	0.350
**FIGO stage**			
**IIIB**	4 (22.2)	0 (0)	
**IIIC**	11 (61.1)	15 (83.3)	0.149
**IV**	3 (16.7)	3 (16.7)	
**Residual disease**			
**None**	6 (33.3)	5 (27.8)	
**<2cm**	5 (27.8)	9 (50.0)	0.277
**>2cm**	7 (38.9)	3 (16.7)	
**Numbers of chemotherapy cycles**			
**<6**	2 (11.1)	3 (16.7)	0.581
**6 or more**	16 (88.9)	14 (77.8)	
**6-month recurrent free survival % (95%CI)**^**b**^	0.73 (0.40-0.91)	0.47 (0.22-0.70)	0.128
**3-year overall survival % (95%CI)**^**b**^	0.30 (0.07-0.58)	0.36 (0.13-0.59)	0.619

^a^ Cut-off = 0.1875, *n* = 36. Percentages may not add to 100% due to missing values of defined variables, missing clinicopathological information was assumed to be missing at random. Variables are indicated as absolute numbers, %, median or range. Age, RFS and OS were evaluated using the Kaplan-Meier method. FIGO stage, residual disease and numbers of chemotherapy cycles were analyzed using the Chi-Square or the Fisher’s Exact test.

^b^*RFS* recurrence-free survival, *OS* overall survival

### Patient characteristics according to CXCR4 positive tumor expression in recurrent ovarian cancer

Twenty-two of forty-three patients showed a high CXCR4 expression, while 21 of 43 patients showed a low CXCR4 tumor expression in recurrent cancer biopsies in the overall cohort. The distribution of the FIGO stage was homogenous between the two groups. Patients with a high dichotomized distribution of CXCR4 positive tumor cells had a significantly higher 6-month RFS rate (*p* = 0.018). The numbers of chemotherapy cycles were also significantly lower in the high CXCR4 expressing group (*p* = 0.006). Finally, the 3-year OS rate was not significantly different between the two groups (*p* = 0.207). Table 3 shows detailed information according to the distribution of high and low CXCR4 tumor expression and clinico-pathological characteristics in recurrent ovarian cancer.

**Table 3:** Patients’ characteristics according to CXCR4 positive tumor expression in recurrent cancer biopsies^a^

	CXCR4 high	CXCR4 low	***p***-value
	***n ***= 22 (100%)	***n ***= 21 (100%)	
**Age (median, range)**	58.5 (34-77)	57 (41-76)	0.458
**FIGO stage**			
**II**	1 (4.5)	0 (0)	
**IIIA**	1 (4.5)	0 (0)	
**IIIB**	2 (9.1)	3 (14.3)	0.715
**IIIC**	13 (59.1)	15 (71.4)	
**IV**	5 (22.7)	3 (14.3)	
**Residual disease**			
**None**	10 (45.5)	5 (23.8)	
**<2cm**	8 (36.4)	7 (33.3)	0.266
**>2cm**	4 (18.2)	8 (38.1)	
**Numbers of chemotherapy cycles**			
**<6**	7 (31.8)	0 (0)	**0.006**
**6 or more**	15 (68.2)	20 (95.2)	
**CS**^**b**^	18 (81.8)	12 (57.1)	0.078
**CR**^**b**^	4 (18.2)	9 (42.9)	
**6-month recurrent free survival % (95%CI)**^**c**^	0.68 (0.45-0.83)	0.48 (0.26-0.67)	**0.018**
**3-year overall survival % (95%CI)**^**c**^	0.50 (0.23-0.72)	0.50 (0.25-0.71)	0.207

^**a**^Cut-off = 84 cells/punch, *n* = 43. Percentages may not add to 100% due to missing values of defined variables, missing clinicopathological information was assumed to be missing at random. Variables are indicated as absolute numbers, %, median or range. Age, RFS and OS were evaluated using the Kaplan-Meier method. FIGO stage, residual disease, numbers of chemotherapy cycles and chemoresistance were analyzed using the Chi-Square or the Fisher’s Exact test.

^b^*CS* chemosensitive, *CR* chemoresistant

^c^*RFS* recurrence-free survival, *OS* overall survival

### Prognostic and predictive significance of pCXCR4/CXCR4 ratio of tumor infiltrating immune cells in primary cancer biopsies

In a Univariate Hazard Cox regression analysis using the dichotomized pCXCR4/CXCR4 ratio expression in primary cancer biopsies there was no significant prediction of a better RFS (HR 0.61; 95CI 0.31 - 1.21; *p* = 0.161). However, the Multivariate Hazard Cox regression analysis showed that a high ratio of pCXCR4/CXCR4 expression (HR 0.33; 95CI 0.13 - 0.81; *p* = 0.015), the presence of residual disease >2cm (HR 7.79; 95CI 2.19 - 27.71; *p* = 0.002) and the number of chemotherapy cycles (HR 1.28; 95CI 1.05 - 1.55; *p* = 0.013) predict RFS independent of FIGO stage, age and number of chemotherapy cycles (Table 4).

**Table 4:** Uni- and multivariate Hazard Cox regression analysis of RFS in primary cancer biopsies^a^

	Univariate			Multivariate		
	HR	95% CI	***p***-values	HR	95% CI	***p***-values
**Age**	1.00	.97 1.03	0.888	1.00	.96 1.04	0.970
**high vs low pCXCR4/CXCR4 TIC ratio**	0.61	.31 1.21	0.161	0.33	.13 .81	**0.015**
**Residual disease <2cm**	1.13	.56 2.28	0.724	1.30	.56 3.03	0.542
**Residual disease >2cm**	3.67	1.62 8.31	**0.002**	7.79	2.19 27.71	**0.002**
**N of chemotherapy cycles**	1.28	1.05 1.55	**0.013**	2.51	.79 8.03	0.120
**FIGO IIIA**	0.34	.02 5.72	0.455			
**FIGO IIIB**	0.93	.11 8.04	0.944			
**FIGO IIIC**	1.21	.16 9.03	0.851	0.63	.17 2.29	0.480
**FIGO IV**	1.48	.18 11.94	0.712	1.07	.24 4.78	0.928

^a^ Cut-off = 0.1875, *n* = 36. Multivariate analyses showing Hazard Ratios and p-value for all primary cancer biopsies (*n* = 35 less than 36 due to missing value) conferred by categorized pCXCR4/CXCR4 TIC ratio in primary cancer biopsies, age, residual disease after cytoreductive surgery, number of chemotherapy cycles and FIGO classification.

### Prognostic and predictive significance of CXCR4 expression in recurrent cancer biopsies

To evaluate the prognostic and predictive significance of CXCR4 and pCXCR4 we calculated the Uni- and Multivariate Hazard Cox regression analysis in primary and recurrent cancer biopsies. Therefore, we generated the Hazard Cox regression considering the dichotomized (p)CXCR4 tumor as well as TIC expression in primary and recurrent cancer biopsies. Finally, the Univariate Hazard Cox regression analysis showed a trend that impact of CXCR4 tumor expression (HR 0.49; 95CI 0.25 - 0.93; *p* = 0.031), the presence of residual disease >2cm (HR 3.67; 95CI 1.62 - 8.31; *p* = 0.002) as well as the number of chemotherapy cycles (HR 1.28; 95CI 1.05 - 1.55; *p* = 0.013) on RFS (Table 5). However, this finding was not independent of known risk factors for recurrence in a Multivariate Hazard Cox regression analysis. There was only a trend for the presence of residual disease >2cm (HR 2.48; 95CI 0.88 - 6.95; *p* = 0.085) in the Multivariate Cox regression analysis.

**Table 5:** Uni- and multivariate Hazard Cox regression analysis of RFS in recurrent cancer biopsies^a^

	Univariate			Multivariate		
	HR	95% CI	***p***-values	HR	95% CI	***p***-values
**Age**	1.00	.97 1.03	0.888	1.00	.97 1.03	0.987
**CXCR4high vs CXCR4low**	0.49	.25 .93	**0.031**	0.57	.24 1.33	0.193
**Residual disease <2cm**	1.13	.56 2.28	0.724	0.81	.36 1.84	0.619
**Residual disease >2cm**	3.67	1.62 8.31	**0.002**	2.48	.88 6.95	0.085
**N of chemotherapy cycles**	1.28	1.05 1.55	**0.013**	1.16	.36 3.73	0.800
**FIGO IIIA**	0.34	.02 5.72	0.455	0.30	.01 6.91	0.449
**FIGO IIIB**	0.93	.11 8.04	0.944	0.64	.07 6.27	0.705
**FIGO IIIC**	1.21	.16 9.03	0.851	0.85	.10 7.08	0.884
**FIGO IV**	1.48	.18 11.94	0.712	0.92	.10 8.22	0.939

^a^ Cut-off = 84 cells/punch, *n* = 43. Multivariate analyses showing Hazard Ratios and p-value for all recurrent cancer biopsies (*n* = 42 less than 44 due to missing value) conferred by categorized CXCR4 expression by tumor cells, age, residual disease after cytoreductive surgery, number of chemotherapy cycles and FIGO classification.

### Spearman Correlation analysis of CXCR4 TIC, CXCR4 tumor expression, pCXCR4 TIC, pCXCR4 tumor expression, MPO, IL-17, FOXP3, OX40 TIC, OX40 tumor expression and CD66b in primary and recurrent ovarian carcinoma

Table 6 illustrates the correlation analysis of different immune markers, CXCR4 and pCXCR4 in primary ovarian cancer. In summary pCXCR4 tumor expression showed a significant correlation with CXCR4 tumor expression (rho = 0.699; *p* < 0.001) and OX40 tumor expression (rho = 0.333; *p* = 0.041). Furthermore, pCXCR4 TIC expression significantly correlated with CD66b (rho = 0.346; *p* = 0.033), while there was no significant correlation for pCXCR4 TIC with CXCR4 TIC (rho = 0.317; *p* = 0.060).

**Table 6:** Spearman correlation analysis in primary ovarian carcinoma

	CXCR4 TIC		CXCR4 tumor	pCXCR4 TIC	pCXCR4 tumor
**CXCR4 TIC**	***rs***	1.000			
	***p***				
**CXCR4 tumor**	***rs***	0.114	1.000		
	***p***	0.458			
**pCXCR4 TIC**	***rs***	*0.317*	0.028	1.000	
	***p***	*0.060*	0.874		
**pCXCR4 tumor**	***rs***	0.104	**0.699**	-0.136	1.000
	***p***	0.546	**<0.001**	0.417	
**MPO**	***rs***	0.078	-0.028	0.194	-0.015
	***p***	0.612	0.857	0.244	0.927
**IL-17**	***rs***	0.038	-0.051	0.077	-0.119
	***p***	0.805	0.741	0.648	0.479
**FOXP3**	***rs***	-0.025	0.140	0.037	0.238
	***p***	0.886	0.409	0.839	0.190
**OX40 TIC**	***rs***	-0.053	0.166	-0.161	0.239
	***p***	0.731	0.277	0.335	0.149
**OX40 tumor**	***rs***	-0.140	*0.271*	-0.056	**0.333**
	***p***	0.361	*0.072*	0.739	**0.041**
**CD66b**	***rs***	0.073	0.004	**0.346**	-0.051
	***p***	0.632	0.978	**0.033**	0.760

Spearman correlation analysis of CXCR4 TIC, CXCR4 tumor expression, pCXCR4 TIC, pCXCR4 tumor expression, MPO, IL-17, FOXP3, OX40 TIC, OX40 tumor expression and CD66b in primary ovarian carcinoma

On the other hand, in Table 7 correlation analyses of different immune markers, CXCR4 and pCXCR4 in recurrent ovarian cancer are shown. These showed the following significant correlations: pCXCR4 TIC expression with CXCR4 TIC expression (rho = 0.394; *p* = 0.019), pCXCR4 tumor expression with CXCR4 tumor expression (rho = 0.507; *p* = 0.002), CXCR4 TIC expression with MPO (rho = 0.577; *p* < 0.001) and CD66b (rho = 0.414; *p* = 0.006), CXCR4 tumor expression with OX40 TIC expression (rho = 0.441; *p* = 0.003) and OX40 tumor expression (rho = 0.576; *p* < 0.001), pCXCR4 tumor expression with OX40 TIC expression (rho = 0.338; *p* = 0.047) and OX40 tumor expression (rho = 0.376; *p* = 0.026).

**Table 7:** Correlation analysis in recurrent ovarian carcinoma

	CXCR4 TIC		CXCR4 tumor	pCXCR4 TIC	pCXCR4 tumor
**CXCR4 TIC**	***rs***	1.000			
	***p***				
**CXCR4 tumor**	***rs***	0.162	1.000		
	***p***	0.299			
**pCXCR4 TIC**	***rs***	**0.394**	0.129	1.000	
	***p***	**0.019**	0.461		
**pCXCR4 tumor**	***rs***	0.035	**0.507**	0.236	1.000
	***p***	0.842	**0.002**	0.172	
**MPO**	***rs***	**0.577**	0.072	0.162	0.146
	***p***	**<0.001**	0.648	0.354	0.402
**IL-17**	***rs***	*0.260*	0.148	-0.074	0.096
	***p***	*0.092*	0.344	0.674	0.583
**FOXP3**	***rs***	0.220	0.160	-0.210	0.057
	***p***	0.203	0.358	0.266	0.764
**OX40 TIC**	***rs***	0.166	**0.441**	0.159	**0.338**
	***p***	0.286	**0.003**	0.360	**0.047**
**OX40 tumor**	***rs***	0.183	**0.576**	0.044	**0.376**
	***p***	0.241	**<0.001**	0.800	**0.026**
**CD66b**	***rs***	**0.414**	0.015	0.138	0.200
	***p***	**0.006**	0.926	0.430	0.249

Correlation analysis of CXCR4 TIC, CXCR4 tumor expression, pCXCR4 TIC, pCXCR4 tumor expression, MPO, IL-17, FOXP3, OX40 TIC, OX40 tumor expression and CD66b in recurrent ovarian carcinoma

### Kaplan Meier survival curves of recurrence-free survival in primary and recurrent cancer

**Fig. 2:**
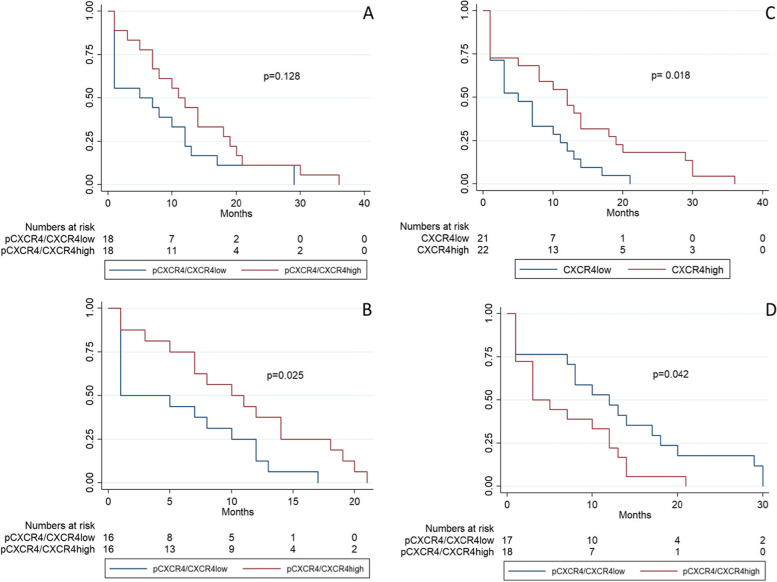
Kaplan Meier survival curve of RFS. Kaplan Meier survival curve of recurrence-free survival according to pCXCR4/CXCR4 TIC ratio in primary cancer (**A, B**) and according to absolute CXCR4 (**C**) as well as relative pCXCR4/CXCR4 (D) tumor expression by tumor cells in recurrent cancer. **A **and **B** Impact of high pCXCR4/CXCR4 TIC ratio expression in primary cancer biopsies on recurrence-free survival (RFS) in patients with high-grade ovarian carcinoma. **B** = early postoperative stage (2-years follow-up). Blue line indicates to tumors with low pCXCR4/CXCR4 TIC ratio and red line refers to tumors with high pCXCR4/CXCR4 TIC ratio. Kaplan-Meier RFS curve was split according to pCXCR4/CXCR4 TIC ratio in patients bearing high-grade ovarian carcinoma as indicated. Cut-off value was 0.1875. **C** Impact of high CXCR4 expression by tumor cells in recurrent cancer biopsies on RFS in patients with high-grade serous ovarian carcinoma. Cut-off value established by using the median of 84 cells/punch. Blue line indicates to tumors with low CXCR4 expression and red line refers to tumors with high CXCR4 expression. **D** Impact of pCXCR4/CXCR4 ratio of tumor expression in recurrent cancer biopsies on RFS in patients with high-grade ovarian carcinoma. Blue line demonstrates tumors with low pCXCR4/CXCR4 ratio of tumor expression, while red line refers to tumors with high pCXCR4/CXCR4 ratio of tumor expression. Kaplan-Meier RFS curve was split according to pCXCR4/CXCR4 ratio of tumor expression in patients bearing high-grade ovarian carcinoma as indicated. Cut-off value was value between high and low pCXCR4/CXCR4 TIC ratio in the overall cohort was set at 0.1875 using the median

Finally, we generated the Kaplan Meier survival curves for primary cancer cases as well as for recurrent cases. Figure 2A illustrates that a high ratio of pCXCR4/CXCR4 tumor infiltrating immune cells in primary cancer biopsies has a positive effect on RFS, nevertheless this is not significant (*p* = 0.126). However, focusing only on the first two years of follow-up the high ratio of pCXCR4/CXCR4 tumor infiltrating immune cells was significantly associated with a better RFS (*p* = 0.025; Fig. 2B). Subsequently, we created the Kaplan Meier survival curve using the association between the absolute CXCR4 expression by tumor cells and RFS. In Fig. 2C, we show that the high expression of CXCR4 by tumor cells is associated with a trend of a better RFS (*p* = 0.018). On the other hand, there was no significant association for OS (*p* = 0.207). While low pCXCR4/CXCR4 ratio of tumor expression in recurrent cancer biopsies in patients with high-grade ovarian carcinoma showed a trend that was associated with better RFS (*p* = 0.042; Fig. 2D).

## Discussion

Over the last couple of years, there has been an increasing scientific interest in the prognostic role of biomarkers in several cancers, especially regarding their outcome prediction and their value as possible new therapeutic targets [3–7]. Several studies have described the potential utility of receptors from the CXC family as biomarkers of response and prognosis in CRC, and CXCR4 expression clearly seems to be a valuable predictor for tumor recurrence in gastrointestinal tumors [33].

Interestingly, very few studies tried to explore the role of CXCR4 and pCXCR4 in HGSOC and their prognostic role in HGSOC is not clear at all. Of note, the phosphorylated form of CXCR4 has not yet been part of a study regarding carcinomas of the ovary [24, 27]. Due to this lack of evidence, we investigated the role of both receptors alone and in relation to each other analyzing tumor expression as well as TIC positivity in a group of HGSOC patients regarding RFS and OS.

In literature, many studies have shown that CXCR4 expression is linked to cancer progression and metastases in hematopoietic as well as in various non-hematopoietic malignancies [15–17]. For instance, a study by Schimanski et al. showed that CXCR4 increases the metastatic potential for different gastrointestinal malignancies (colorectal, pancreas and liver). Furthermore, CXCR4 expression might be a valuable predictor for tumor recurrence in lung cancer [33].

Given the importance of CXCR4 as a negative prognostic factor in several cancers, our group also studied the role of the phosphorylated, activated form of CXCR4 (pCXCR4) as a prognostic factor in CRC. In summary, Weixler et al. showed for the first time that the expression of the activated form of CXCR4 on tumor cells represents a favorable prognostic factor in CRC [28]. A recent study also analyzed the role pCXCR4 in a lymphoblastic leukemia population which, contrary to colorectal cancer, showed an association of pCXCR4 with poor survival in adult patients with B-acute lymphoblastic leukemia [21].

These results in solid tumors also differ from the results of a meta-analysis by Liu et al., which showed that high CXCR4 expression was associated with poor prognosis in HGSOC. Reviewing these results, we noted that high CXCR4 expression in tumors from Asian but not European populations was statistically associated with poor prognosis [34]. Other studies also investigated the prognostic significance of CXCR4 in HGSOC. Sekiyi et al. confirmed that expression of CXCR4 indicates poor prognosis in patients with OC, but this was in clear cell carcinoma of the ovary [27], a pathogenically different subtype.

To better understand our results we also focused the review of the literature on the CXCL12-CXCR4 axis, as a potential therapeutic target [13, 35–37]. Wang et al. has shown that CXCR4 is highly expressed in malignant ovarian tumors and ovarian cancer cell lines, and different expression of CXCR4 was observed between ovarian cancers with or without lymph node metastasis. This group also demonstrated that knockdown of CXCR4 reduces proliferation and invasion of ovarian cancer cells through modulating the Wnt/β-catenin pathway [38].

The interference of chemotherapeutics with the CXCL12-CXCR4 axis might explain why patients in our study with overexpression of CXCR4 in recurrent cancer biopsies have a smaller number of chemotherapy cycles because CXCR4 is one of the key molecules in the mechanism of action of cisplatin-based chemotherapy. Although Li et al. has shown that patients with a higher CXCR4 expression had a significantly lower chemosensitivity, another study showed that knockdown of CXCR4 by small interfering RNA suppressed cell proliferation and resulted in G1/S arrest, increased apoptosis and increased chemosensitivity in both cisplatin-sensitive A2780 cells and cisplatin-resistant A2780/cis cells in an in-vitro OC cell model [39]. Mao et al. demonstrated that CXCR4 has been significantly correlated with the progression of HGSOC and that silencing of CXCR4 to limit SDF-1/CXCR4 signaling greatly suppressed tumor growth by reducing cell proliferation [37].

In our study, we observed that a high ratio of pCXCR4/CXCR4 TIC in primary cancer cases was associated with a better RFS, especially during the early follow-up phase (first 2 years). Interestingly, this finding was even independent of known risk factors such as age, FIGO stage and residual disease for recurrence in a Multivariate Cox regression analysis. Overall our results indicate that during the early postoperative period the immune response plays the most important role to control the progression of the disease. The Kaplan Meier survival curves showed that the pCXCR4/CXCR4 tumor infiltrating immune cells in primary cancer biopsies have a tendential positive effect on RFS, nevertheless this was not significant (*p* = 0.126). However, focusing on the first two years of follow-up the high ratio of pCXCR4/CXCR4 tumor infiltrating immune cells showed a trend that was associated with a better RFS (*p* = 0.025; Fig. 2B). This could be evidence that at the beginning of the disease the immune response plays a crucial role in the fight against cancer cells. On the other side, we observed a trend that association between high CXCR4 tumor expression in recurrent cancer biopsies and better RFS, what might indicate that in recurrent disease the biology of the tumor itself is the most crucial factor for prognosis and not the immune response. Finally, we noticed that a high ratio of pCXCR4/CXCR4 tumor expression was associated with a worse RFS in the Kaplan Meier survival curves of recurrent cancers which most likely mirrors the fact, that in recurrent disease the expression of CXCR4 is the dominant factor and is associated with a certain biological behavior of the tumor. Therefore, we hypothesize that in recurrent HGSOC the biology of the tumor is more important than the immune response to control tumor progression. Finally, high CXCR4 tumor expression in recurrent HGSOC biopsies might be indicative for sensitivity to repeated chemotherapy.

Although the exact molecular mechanism needs to be further evaluated, the results of our study are important as we could show that the activated form of CXCR4 (pCXCR4) in relation to the non-activated one on TIC significantly predicted a better RFS independent of known risk factors for recurrence in primary cancer. Overall, our results indicate that in primary cancer the immune response plays an important role for disease control, especially during the early postoperative stage whereas in recurrent cancer CXCR4 tumor expression seems to mirror a certain tumor biology rather than having a functional role concerning chemosensitivity in HGSOC. Finally, our results are in line with most previous studies showing that the prognostic value of high pCXCR4 to be superior to that of high CXCR4 [21, 28, 29]. Regulation of phosphorylation and internalization has significant effects on CXCR4-mediated cellular responses. Furthermore, our results on tumor expression of pCXCR4 not being significantly associated with RFS might not be due to a functional problem but more likely lies in limited DNA sequencing of tumor biology. That is why we believe that our results are of clinical interest. Next generation sequencing and RNA-sequencing might shed new light on the biological role of CXCR4.

Our study also has several limitations. First, even though the cohort is well characterized, the results are based on a retrospective analysis and the patient cohort is small. This study illuminates only an interesting and potentially relevant predictive/prognostic marker for ovarian cancer patients. We are currently conducting a prospective study with a larger cohort to validate these results. Secondly, scoring has been done on only one punch biopsy. Multiple biopsy samples would have been favourable to avoid sampling bias. On the other hand, we explored for the first time the predictive and prognostic role of pCXCR4 in relation to CXCR4 in high grade serous ovarian cancer.

## Conclusion

Our study shows for the first time that a high pCXCR4/CXCR4 TIC ratio in primary HGSOC biopsies is indicative for better RFS and response to chemotherapy.

## Data Availability

The datasets used and analyzed during the current study are available from the corresponding author on reasonable request.

## Abbreviations

CI: Confidence intervals
CRC: Colorectal cancer
EOC: Epithelial ovarian cancer
HGSOC: High-grade serous carcinomas
HR: Hazard ratio
IHC: Immunohistochemistry
MPO: Myeloperoxidase
OC: Ovarian Cancer
OS: Overall survival
RFS: Recurrent free survival
TIC: Tumor infiltrating immune cells
TMA: Tissue microarrays
